# Predictions on Structural and Electronic Properties to Synthesize Bismuth-Carbon Compounds in Different Periodicities

**DOI:** 10.3390/ma15228150

**Published:** 2022-11-17

**Authors:** Abdul Majid, Tariq M. Younes, Alia Jabeen, Hira Batool, Mohammad Alkhedher, Sayed M. ElDin

**Affiliations:** 1 Department of Physics, University of Gujrat, Gujrat 50700, Pakistan; 2 Department of Mechatronics Engineering, Faculty of Engineering Technology, Al Balqa Applied University, Amman 1705, Jordan; 3 Mechanical and Industrial Engineering Department, Abu Dhabi University, Abu Dhabi 111188, United Arab Emirates; 4 Center of Research, Faculty of Engineering & Technology, Future University in Egypt, New Cairo 11835, Egypt

**Keywords:** BiC compounds, density functional theory, structural properties, electronic properties, unit cell, supercell

## Abstract

This work was carried out to explore the compounds of bismuth with carbon using density functional theory (DFT)-based computations. The structures of the compounds BiC, BiC_2_, BiC_3_, Bi_2_C_3_, BiC_5_, and Bi_2_C_5_ were predicted at a generalized gradient approximation (GGA-PBE) level of theory. The calculations were carried out on the structures in unit cell and supercell geometries in slab and bulk periodicities. The structural and electronic properties of the mentioned compounds were investigated in detail. The calculations of the structures revealed lattice constants of the compounds for cubic unit cell as 212.2 pm for BiC, 176.9 pm for BiC_2_, 240.5 pm for BiC_3_, 232.4 pm for Bi_2_C_3_, and 354.5 pm for Bi_2_C_5_. The compounds BiC, BiC_2_, BiC_3_, BiC_5_, and Bi_2_C_5_ were found to be metallic, whereas Bi_2_C_3_ exhibited semiconducting character with a band gap of 0.305 eV. This work provides an initial framework for preparing new 2D materials from Bi_x_C_y_.

## 1. Introduction

The theoretical predictions to synthesize new materials have been on prime focus in computational material science [1,2,3,4,5,6,7]. The preparation of metal-carbon compounds has recently attained significant research interest, due to their far-reaching applications [8,9]. There are several compounds of Bi that have been reported and found suitable for different applications. An orthorhombic crystal system Bi_2_S_3_ in one dimension has been reported to be an n-type semiconductor material with a band gap of 1.2 eV and found suitable for optoelectronic devices [10]. Bi_2_Se_3_ is a 2D material belonging to the P-3m1 space group having a band gap of 1.42 eV and, hence, can be used for optoelectronic, photo catalytic, and photovoltaic applications [11]. Bi_2_Se_3_ shows the topological insulating behavior, which can be tuned by strain in the structure [12]. The bulk BiI_3_ with hexagonal structure has been reported with a band gap 1.57 eV [13]. BiP is a 2D material with a band gap of 0.92 eV having applications in ferro-electric gadgets [14]. BiP_3_ is also a 2D material having monoclinic and trigonal symmetries with a band gap of 1.36 eV, which is favorable for photo-catalytic usage [15]. InBi is a 2D material with a band gap 0.35 eV, whose structural symmetry shows a resemblance to hexagonal lattice [16]. BiOX (X = Br, I, Cl) is a class of materials in which BiOI and BiOCl are 3D, while BiOBr has 2D periodicity with band gap in ranges from 1.85 eV to 3.44 eV [17]. PdBi_2_ is a 2D material that has a tetragonal symmetry with a band gap of 1.53 eV and was found suitable for catalytic application [18]. The series of BiX compounds (X = H, F, Cl, and Br) are 2D topological insulators with a hexagonal symmetry and band gap values in the range of 0.32–1.08 eV [19].

The compounds of bismuth with non-metals and compounds of carbon with metals in different periodicities are available in the literature. The compounds of bismuth with other elements include Bi_2_S_3_, Bi_2_Se_3_, BiI_3_, BiN, BiP, BiP_3_, InBi, BiOX (X = Br, I, Cl), PdBi_2_, and BiX (X = H, F, Cl, and Br). Bi_2_S_3_ appeared orthorhombic in bulk with lattice parameters 1.112 nm, 1.125 nm, and 0.397 nm, whereas its band gap was 1.2 eV, which points to its applications in FETs and photodetectors [20]. BiI_3_ appears in bulk with a hexagonal symmetry, and its investigations revealed an indirect band gap of 1.57 eV using spin-orbit coupling that permits the usage of the material for photonics and photovoltaic cells [21].

The famous carbon compounds include AlC, BC, Be_2_C, C_3_N, CSe, SiC, Fe_3_C, Fe_5_C_2_, Fe_2_C, etc. BC is graphene-like material having a lattice constant of 2.46 Å with metallic conductivity [22]. Be_2_C is also a 2D material with a pentagonal symmetry having a lattice constant of 3 Å and direct band gap of 1.65 eV [23]. The slabs of C_3_N are a 2D material with lattice parameters of 4.86 Å, which is reportedly changed by applying the biaxial and uniaxial strains [24]. Its DFT-calculated structural properties indicated a strain-dependent bond length of 1.403 Å, whereas the electronic properties revealed an indirect band gap, 0.39 eV, which almost matched with the earlier theoretical findings [25]. CSe is a 2D material that has been studied using DFT calculations at the GGA-PBE level of theory [26]. The electronic property results showed that the CSe is a direct semiconductor with a band gap of 0.9 eV, which predicted its usage in optoelectronics [27]. The compounds of Fe with C include cementite (Fe_3_C), Hägg (Fe_5_C_2_), and eta-carbide (η-Fe_2_C) [28]. The structures of Fe_3_C and η-Fe_2_C are orthorhombic, whereas Fe_5_C_2_ has a monoclinic structure. The calculations performed on SiC sheets indicated that the bond length Si-C is 1.649 Å for 1D [29]. The 2D SiC has a honeycomb symmetry with lattice parameters of 3.094 Å and a Si-C bond length of 1.786 Å [30]. The 3D SiC has a cubic symmetry with lattice parameters of 3.096 Å and a bond length of 1.907 Å [31]. The electronic properties reveal that 2D SiC material is a semiconductor with a band gap of 2.53 eV, while in 3D, the band gap is 1.41 eV. Carbon has compounds with several transition metals, such as Ta_2_C, Ta_3_C_2_, Ta_4_C_3_, Ti_2_C, Ti_3_C_2_, Ti_4_C_3_, V_2_C, Cr_2_C, Zr_2_C, and Hf_2_C [32].

Bi_2_C_3_ is the only reported compound of Bi and C in which sp^2^ hybridization occurs in C atoms, and the three-fold coordination of lone pair electrons was found for Bi [33]. The structure of the 2D monolayer of Bi_2_C_3_ was observed to be of honeycomb shape and has a lattice parameter of 6.70 Å. The monolayers of Bi_2_C_3_ are intrinsic semiconductors with band gaps of 0.81 eV. For Bi_2_C_3_ sheets, the electrons are confined around the Bi atom, and the bond C–C, suggests that the C atom receives supported planar sp^2^ hybridization, and the Bi atom leans toward non-planar sp^3^ hybridization with lone pair electrons [34]. The partial density of states (PDOS) indicate that the edge of the valence band (VBM) is dominated by the p orbitals of Bi atom hybridized with noticeable contribution from the C-p orbitals, while the conduction band edge (CBM) originates from the C-p orbitals and somewhat from the Bi-p orbitals. The effects of strain on the band structures of 2D Bi_2_C_3_ films have been studied [33]. The findings indicated that CBM and VBM linearly depend on strain, and optical absorption in Bi_2_C_3_ occurs in the UV and visible regions, which indicates the productive usage of the material for optical devices.

Considering the abundance of carbon and its potential to make compounds with different elements, the literature has shown a rich list of such compounds. A detailed literature survey pointed out that the compounds of bismuth with carbon have not been reported so far. This study is dedicated to the computational design of compounds of Bi with carbon using density functional theory (DFT), which provides an efficient framework to predict new materials [35,36]. The compounds of Bi and C studied in this work include BiC, BiC_2_, BiC_3_, Bi_2_C_3_, BiC_5_, and Bi_2_C_5_, whose structural and electronic properties are investigated in detail using unit cell and super cell approaches.

## 2. Computational Detail

The entire calculations were based on DFT computations, which were carried out using the linear combination atomic orbital (LCAO) scheme employed in ADF-BAND code [37]. The Bi-C compounds reported herein have not been reported yet, due to which, no structure files are available. These structures were manually prepared starting from simple BiC unit cell to successively extending to increase in Bi and C atoms in the structures by allowing for complete structural relaxation. The unit cell of BiC contains one Bi and one C atom, while the 2 × 2 super cell of BiC contains four Bi atoms and four C atoms. The unit of BiC_2_ contains 1 Bi atom and 2 C atoms, while the 2 × 2 super cell slab of BiC_2_ contains 8 Bi atoms and 16 C atoms. The unit of BiC_3_ contains 1 Bi atom and 3 C atoms, while 3 × 3 super cell of BiC_3_ contains 8 Bi atoms and 24 C atoms. The unit of Bi_2_C_3_ contains 2 Bi atoms and 3 C atoms, while the 2 × 2 super cell of Bi_2_C_3_ contains 8 Bi atoms and 12 C atoms. The unit of Bi_2_C_5_ contains 2 Bi atoms and 5 C atoms, while the 3 × 3 super cell of Bi_2_C_5_ contains 18 Bi atoms and 45 C atoms. The 2 × 2 super cell of BiC_5_ contains 9 Bi atoms and 45 C atoms.

The geometries of the unit cells and supercells were fully optimized to obtain relaxed structures. The maximum Cartesian step allowed for the convergence of geometry is 1 × 10^−3^ Å. The convergence criteria for energy was 10^−5^ eV. The maximum RMS gradient allowed was 6.66 × 10^−4^ Hartree/Å. For the optimizations of structure, the quasi-Newton model was used. The optimized parameter considered in this method is the Hessian update method, which is based on the BFGS (Broyden–Fletcher–Goldfarb–Shanno) algorithm. All the basic functions were confined to a radius of 10 Bohr. The calculations were carried out at a generalized gradient approximation with Perdew–Burke–Ernzerhof (GGA-PBE) level of theory [38] and triple zeta polarization (TZP) basis sets were employed. In order to investigate the bi-layered materials, D3 correction offered by Grimme was included to count the interlayer van der Waals interactions [39]. Considering presence of Bi, the scalar relativistic effects ZORA (zeroth order regular approximations) were included.

## 3. Results and Discussion

The results obtained to investigate the structural and electronic properties of the entire series of Bi-C compounds are described below.

### 3.1. Structural Properties

**(A)**  **Unit Cell of BiC**

**Figure 1:**
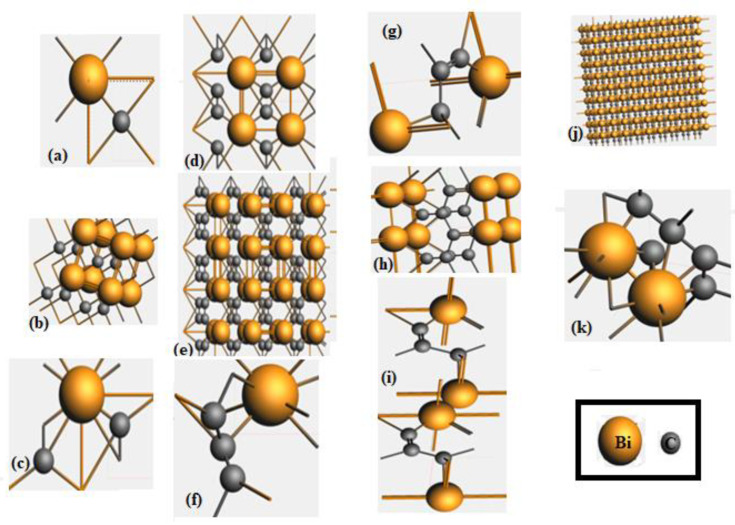
The optimized structure viewed along z-axis for (**a**) BiC unit cell (**b**) BiC supercell (**c**) BiC_2_ unit cell (**d**) BiC_2_ slab (**e**) BiC_2_ supercell (**f**) BiC_3_ unit cell (**g**) Bi_2_C_3_ unit cell (**h**) Bi_2_C_3_ supercell (**i**) Bi_2_C_3_ bilayer (**j**) Bi_2_C_3_ monolayer (**k**) Bi_2_C_5_ unit cell. The orange spheres represent Bi, whereas gray spheres indicate C atoms. The single, double, and aromatic bonds (with dotted lines) between Bi and C atoms are shown.

The converged geometry of the BiC unit cells in 2D slab and 3D bulk periodicity is given in Figure 1. In case of 2D structure, the Bi-C bond length appeared as 212.2 pm, whereas the bond energy was equal to −6.41 eV. The Hirshfeld charge analysis revealed that the net atomic charge on Bi was 0.321 e, and on C it was −0.321 e. On the other hand, the bond length Bi-C for 3D structure was found to be 212.1 pm. The computed results reveal that bond energy was −6.39 eV, the Hirshfeld charge on Bi was 0.316 e and on C was −0.316 e. The computed values pointed out that the charge on Bi appeared positive, whereas it was negative on C atoms. This finding points to the fact that Bi appeared cationic, whereas C was anionic in nature in the Bi_x_C_y_ compounds, which agrees with the literature [40]. The transfer of charge from the Bi to C atoms, in the case of Bi-doped graphene, has been reported [41].

**(B)**  **Super Cell of BiC**

The optimized geometry of the BiC supercell is shown in Figure 1. In the case of 2D, the values of the bond lengths Bi = Bi and Bi-C appeared as 300 pm and 212.2 pm, respectively, whereas the dihedral angles C-Bi-C or Bi-C-Bi were 90° and Bi-Bi-C is 45°. The bond energy for this material was −81.66 eV, whereas the Hirshfeld charges on Bi and C were 0.214 e and −0.214 e, respectively. On the other hand, in the case of the 3D supercell of BiC, the values of the bond lengths appeared as Bi=Bi at 300 pm in the intra-layer with bond order 2.0 and Bi-Bi at 359 pm for the interlayer. The bond length Bi-C was 226.3 pm with bond orders of 1 and 1.5, pointing to an aromatic bond, whereas the C-C bond length was 331.2 pm. The value of the dihedral bond angle varied from 89.7° to 89.8° for the Bi-C-Bi arrangement of atoms. The bond energy for this material was −52.29 eV, and the Hirshfeld charges on Bi and C were 0.327 e and −0.327 e, respectively. The computed values pointed out that the charge on Bi appeared to be positive, whereas it was negative on the C atoms. This finding points to the fact that Bi appeared to be cationic, whereas C was anionic in nature, which agrees with the literature [40].

**(C)**  **Unit Cell of BiC_2_ in 2D and 3D**

The unit cell of BiC_2_ is shown in Figure 1. The computed values of bond length Bi-C for the 2D unit cell was between 176.9 pm for one carbon atom and 254.4 pm for the other. The bond angle between the Bi and C in arrangement of Bi-C-Bi was 93.8°. The Hirshfeld charge analysis showed that the net atomic charge on Bi was 0.515, and it was −0.257 e on both C atoms. In case of the BiC_2_ unit cell in the 3D the bond length, Bi-C was 176.7 pm for one carbon and 254.6 pm for the other one. The bond angles in arrangements such as C-Bi-C and Bi-C-Bi were 90.5° and 53.8°, respectively. The Hirshfeld charge analysis show that the net charge on Bi was 0.510, while on every C atom its value was −0.255 e. The computed values pointed out that the charge on Bi appeared positive, whereas it appeared negative on C atoms. This finding points to the fact that Bi appeared cationic, whereas C was anionic in nature, which agrees with the literature [40].

**(D)**  **Super Cell of BiC_2_ in 2D and 3D**

We designed multiple super cells of BiC_2_ as 2 × 2 slab, 2 × 2 bilayer-slab, 4 × 4 monolayer slab, and 4 × 4 bilayer slab. The computed results revealed that the bond length Bi=Bi was 300 pm, with bond orders of 2.0 and 3.0, whereas the values of the bond lengths of C-C were 145.4 pm and 150 pm, with a bond order 1.0. The bond length Bi-C is in range 409–409.2 pm, whereas dihedral bond angles of Bi-Bi-Bi and C-C-C were 90° and its 79.4°, respectively. In the case of an optimized structure of a BiC_2_ super cell, the results show that the bond length Bi-Bi was 300 pm with bond order 1.0, whereas Bi-C was 254.8 pm with the bond order 1.0. The bond angle of Bi-Bi-Bi was 90°, and Bi-C-Bi was 45.8°. The Hirshfeld charges on the Bi and all C atoms were 0.541 e and −0.270 e, respectively. The computed values pointed out that charge on Bi appeared positive, whereas it was negative on C atoms. This finding points to the fact that Bi appeared cationic, whereas C was anionic in nature, which agrees with the literature [40].

In case of the 4 × 4 monolayers in 2D and 3D, the values of the bond lengths, bond orders, and bond angles appeared to be the same. The bond angle of the Bi-Bi was 300 pm with bond order 1.0, whereas the bond lengths of Bi-C were 177.2 pm and 245.6 pm. The bond length C-C was 111 pm with bond order 1.0. The bond angle Bi-C-Bi was 115.7°, whereas the bond angle Bi-C-C or C-C-Bi was found to be 122.2°. The bond angle Bi-Bi-C or C-Bi-Bi was 32.2°. The Hirshfeld charge on Bi and C was 0.549 e, and C was −0.274 e. The computed values pointed out that charge on Bi appeared positive, whereas it was negative on the C atoms. This finding points to the fact that Bi appeared cationic, whereas C was anionic in nature.

In the case of the 4 × 4 bilayer, the bond angle Bi-Bi was 300 pm with the bond orders 1, 1.5, and 2. The bond length Bi-Bi was also 524.8 pm, whereas Bi-C appeared as 177.2 pm and 245.6 pm. The bond length C-C was 111.4 pm with bond order 1.0. The bond angle of Bi-Bi-Bi was 90°, whereas its value for Bi-C-C or C-C-Bi appeared as 122.2°. The bond angle also had a value of 57.8° in the Bi-C-C arrangement.

In case of the 4 × 4 bilayer in the slab, the bond angle, bond order, and bond angle were almost same for both layers. The bond of Bi-Bi was 300 pm with bond orders such as 1.5, 2, and 3. The bond lengths for Bi-Bi were found to be 714.2 pm and 804.5 pm, while the bond lengths for Bi-C were 285.2 pm and 286.3 pm with a bond order of 1.5. The bond lengths between C-C were 157.5 pm and 142.5 pm with bond order 1.5. The bond angle Bi-Bi-Bi was 90°, while its values for Bi-C-C or C-C-Bi were found to be 73.6°, 105.9°, and 106.1°. The bond energy of the 4 × 4 bilayer slab material was −532.49 eV.

**(E)**  **Unit cell of** **BiC_3_**

The optimized structure of the unit cell of BiC_3_ is given in Figure 1. The unit cell of BiC_3_ in 2D exhibited bond lengths for Bi-C of 240.5 pm and for C-C of 143.5 pm. The bond angle between the Bi and C in the arrangement of Bi-C-C was 131.1°, and for C-C-C the bond angle was 57.2°. The bond energy for this material was −24.53 eV, whereas Hirshfeld charge analysis revealed that the net atomic charge on Bi was 0.132 e, while on one C its value was −0.142 e. The computed values pointed out that the charge on Bi appeared to be positive, whereas it was negative on C atoms. This finding points to the fact that Bi appeared cationic, whereas C was anionic in nature, which agrees with the literature [40,41]. In the case of the BiC_3_ unit cell in 3D, the bond length Bi-C was 226.6 pm, with a bond order of 1.0, while a bond length of C-C was 133.3 pm. The bond angles Bi-C-C had values of 84.3° and 120.1°, while the bond angle of C-C-C was 90.9°. The bond energy of the material was −84.61 eV.

**(F)**  **Super Cell of BiC_3_**

We designed multiple super cells of BiC_3_. In the case of the super cells of BiC_3_ in 2D, the computed results revealed that the values of bond length Bi=Bi were in the range of 299.6–309.4 pm with bond orders of 2.0 and 3.0. The bond length C-C was in the range of 138.1–138.2 pm with a bond order of 1.5. The bond angle Bi-Bi-Bi had values of 86.6°, 88°, and 88.2°, while the C-C-C values were 55.8°, 124.1°, and 124.3°. The values of the dihedral angles of Bi-C-C were 145.7°, 145.9°, and 146°, while C-C-C had values of 90°, 90.1°, 118.8°, 118.9°, and 151.3°. The bond energy of this material was −222.96 eV. In the case of the super cell of BiC_3_ in 3D, the computed results revealed that the bond length Bi=Bi was 300 pm, while the values of bond length C-C were 133.3 pm and 134.7 pm with a bond order 1.0. The bond length of Bi-C varied by values such as 207 pm, 2214 pm, 237.7 pm, and 246.9 pm with a bond order of 1.0. The bond angle Bi-C-C had the values 60.3°, 77.9°, and 90.3°, whereas the value for C-C-C appeared to be 99.9°.

**(G)**  **Unit Cell of Bi_2_C_3_**

The unit cell of Bi_2_C_3_ was modeled in 2D and 3D. In the case of the 2D unit cell, Bi-C had bond lengths values of 218.9 pm and 232.4 pm. The bond length C-C was 134.8 pm for one C atom and 170.4 pm the other C atom. The bond angles Bi-C-Bi had two values, 110.5° and 136.7°, while C-C-C had a value of 115°. The bond energy was −28.45 eV, whereas the Hirshfeld charges on two Bi atoms were 0.121 e and 0.161 e, while on C, the charge values were −0.018 e, −0.091 e, and −0.173 e. The computed values pointed out that charge on Bi appeared positive, whereas it appeared negative on the C atoms. This finding points to the fact that Bi appeared cationic, whereas C was anionic in nature. In the case of the 3D unit cell of Bi_2_C_3_, the value of bond length Bi-Bi was 212.6 pm, while bond length Bi-C had five different values, i.e., 180.1 pm, 198 pm, 198.1 pm, 226.7 pm, and 283.1 pm. The bond length C-C was 179.7 pm with the bond order 1.0. The bond angle Bi-Bi-C had three values, 66.9°, 91.9°, and 120.9°. The bond angle for C-C-Bi had a value of 63°, while its value for Bi-C-C was 56.1°. The Hirshfeld charge analysis shows that the net atomic charge on two Bi were the same, i.e., 0.464 e, and two C atoms had the same charge, i.e., −0.313. e, and on the third carbon, it was −0.302 e.

**(H)**  **Super Cell of Bi_2_C_3_**

The optimized structure of a super cell of Bi_2_C_3_ in 2D is shown in Figure 1. The computed results revealed that the bond length of Bi-Bi was 300 pm with bond orders of 2.0 and 3.0. The bond length of Bi-C was 228.6 pm when the bond order was 2, whereas for a bond order of 1.5, its value was 244.2 pm. The bond length C-C has three values, 300 pm, 135.2 pm, and 163.8 pm, with a bond order of 1.0. The bond angle in the arrangement of Bi-Bi-C or C-Bi-Bi had two values, which were 52.6° and 90.3°, while for the arrangement Bi-C-C or C-C-Bi, it had three values, which were 52°, 113.2°, and 113.5°. The bond angles in an arrangement such as C-C-C had four values, which were 87.3°, 89.7°, 92.7°, and 113.3°. The bond energy for this material was calculated to be −121.19 eV. The Hirshfeld charge analysis revealed that the net atomic charges on all Bi were 0.080 e, 0.080 e, 0.080 e, 0.080 e, 0.132 e, 0.132 e, 0.132 e, and 0.132 e, while on all C atoms, the values were −0.065 e, −0.065 e, −0.065 e, −0.065 e, −0.030 e, −0.030 e, −0.030 e, −0.030 e, −0.116 e, 0.116 e, 0.116 e, and 0.116 e. The computed values pointed out that the charge on Bi appeared to be positive, whereas it appeared to be negative on the C atoms. This finding points to the fact that Bi appeared cationic, whereas C was anionic in nature, which agrees with the literature [40,41].

The super cell of Bi_2_C_3_ was also optimized in 2D for monolayer and bilayer slabs. The computed results revealed that the bond length of Bi-Bi was 394.1 pm. The bond length of Bi-C had four values, which were 218.8 pm, 221.3 pm, 233.8 pm, and 234.2 pm. The bond length of C-C with bond order 2 had two values, which were 134.3 pm and 134.4 pm. The other values of C-C included 164.3 pm and 181.9 pm. The bond angles of Bi-C-C and C-C-Bi had the values 108.8°, 109.8°, 136.9°, and 137°. Their bond energy was −57.57 eV. The Hirshfeld charge analysis showed that the net atomic charges on Bi were 0.121 e, 0.156 e, 0.120 e, and 0.164 e, and on C, the charges were −0.018 e, −0.091 e, −0.168 e, −0.017 e, −0.094 e, and −0.173 e. The computed values pointed out that the charge on Bi appeared to be positive, whereas it appeared to be negative on C atoms. This finding points to the fact that Bi appeared cationic, whereas C was anionic in nature.

Now, we discuss the 10 × 10 mono-layered and bi-layered slabs of Bi_2_C_3_ in 2D. First, we discuss the 10 × 10 slab of Bi_2_C_3_, for which the computed results revealed the bond length of Bi-Bi as 300 pm, with bond orders of 1.5, 2, and 3. The bond length of Bi-C had two values, which were 215.3 pm and 226.7 pm, with a bond order of 1.0. The bond length of C-C with a bond order of 2 had a value of 132.6 pm, while the other that had a bond order of 1.0 had the values 166.5 pm and 166.6 pm. The bond angle Bi-Bi-Bi had the value 90°, while Bi-C-C or C-C-Bi had the bond angle values 110.5°, 111.8°, and 136.7°. The bond angle of Bi-C-Bi had the value 86.5°, whereas the bond angle for C-C-C had three values, which were 115°, 11.8°, and 128.1°.

In the case of the bi-layered slab of Bi_2_C_3_, the computed results revealed that the bond lengths of Bi-Bi, Bi-C and C-C in one slab had the same value as mentioned in the above paragraph. The bond length of Bi-Bi was 689.1 pm, whereas the values of the bond lengths of Bi-C were 854.9 pm, 855 pm, 936.2 pm, and 936.3 pm. The bond angle Bi-Bi-Bi had the value 90°, while Bi-C-C or C-C-Bi had the bond angle value 109.4°. The bond angle of Bi-C-Bi had the value 88.3°, while the bond angle for C-C-C had the values 115.8° and 128.4°.

**(I)**  **Super Cell of BiC_5_**

The computed results on the optimized super cells of BiC_5_ revealed that the bond length of Bi-Bi was 300 pm with the bond orders 2 and 3, whereas the bond length of Bi-C had two values 245.1 pm and 365.5 pm with bond order 1.0. the difference in behavior is due to the fact when the slab is thin then the behavior of every Bi is different towards Carbon atoms [42]. The bond length C-C with bond order 1 had three values, which were 142.7 pm, 159.8 pm, and 170.7 pm, while the others that had bond orders of 1.5 and 2 had the values 136.7 pm with bond order 2 and 139 pm and 140.7 pm with the bond order 1.5. The bond angle Bi-Bi-Bi had the value 90°, while Bi-C-C had the bond angle values 95.3°, 100.1°, 134.1°, and 134.3°. The bond angle for C-C-C had eight values, which were 91.4°, 105.1°. 111.2°, 113.6°, 114.8°, 130.2°, 130.4°, and 131.2°. The bond energy for this material was −361.31 eV. The Hirshfeld charge analysis showed that the net atomic charge on all Bi was the same, which is 0.085 e, and the values of the charge on C were found, i.e., −0.017 e, −0.008 e, −0.027 e, −0.030 e, and −0.037 e. The computed values pointed out that the charge on Bi appeared positive, whereas the charge appeared negative on C atoms. This finding points to the fact that Bi appeared cationic, whereas C appeared anionic in nature.

**(J)**  **Unit Cell of Bi_2_C_5_**

The unit cell of Bi_2_C_5_ was optimized in 2D and 3D. In the case of the 2D unit cell of Bi_2_C_5_, the computed result showed that the bond length Bi-C was 354.5 pm. The bond length of Bi-C had five values, which were 255.4 pm, 347.5 pm, 361.7 pm, 499.8 pm, and 501 pm. The bond length of C-C had three values, in which 159.pm and 146.6 pm appeared with bond order 1 and 138.1 pm appeared with the bond order of 1.5. The bond angles of Bi-Bi-C and C-Bi-Bi had the value 109.4°. The bond angles Bi-C-C and C-C-Bi were 81.3°, 117.1°, and 119.8° whereas the values for C-C-C were 111.8°, 112°, and 128.8°. The bond energy for this material was −41.53 eV. The Hirshfeld charge analysis showed that the net atomic charges on Bi were 0.160 e and −0.021 e, and on the C atom, the values were −0.057 e, 0.005 e, −0.059 e, −0.032 e, and 0.003 e. In the case of Bi_2_C_5_ in the 3D bond length, Bi-B was 212.1 pm. The bond length values of Bi-C were 177.5 pm, 193.4 pm, and 212 pm. The bond angles of Bi-C-C and C-C-Bi had values 56.2°, 78.4°, 79.5°, and 86°, while bond angle C-C-C had values of 45°, 90°, 119.4°, and 179.9°. The bond energy of the material was found to be −84.61 eV.

**(K)**  **Super Cell of Bi_2_C_5_ in 2D**

The super cell of Bi_2_C_5_ was optimized in 2D, as shown in Figure 1. The computed results revealed that the bond length Bi-Bi had three values, which were 300 pm with the bond order 1.5, and 419.6 pm and 323.8 pm with bond order 3. The bond length of Bi-C had the value 260.5 pm with bond order 1.0. The bond length of C-C with bond order 1 had four values, which were 143.1 pm, 146.6 pm, 159.2 pm, and 170.6 pm, while the other that had bond orders of 1.5 and 2 had the values 139.7 pm and 140.6 pm with bond order 1.5 and 137.2 pm with the bond orders 1.5 and 2. The bond angle in the arrangement of Bi-Bi-Bi had the values 89.4° and 90°. Bi-C-C had bond angle values of 101.1°, 131.8°, and 133°. The bond angles for C-C-C had six values, which were 92.4°, 113°, 115.4°, 130°, 130.8°, and 130.9°. The bond energy for this material was −391.96 eV. The Hirshfeld charge analysis showed that the net atomic charge on first nine Bi was 0.090 e, while on C atoms, the charge values were −0.013 e, −0.001 e, −0.028 e, −0.026 e, and −0.038 e. The computed values pointed out that charge on Bi appeared positive, whereas it appeared negative on the C atoms. This finding point to the fact that Bi appeared cationic, whereas C was anionic in nature, which agrees with the literature [40,41]. The optimized values of the lattice constant, angles, area of slabs, volume of bulk cells, and structural symmetry of the studied compounds are given in Table 1.

### 3.2. Electronics Properties

**(A)**  **Unit Cell of BiC**

**Figure 2:**
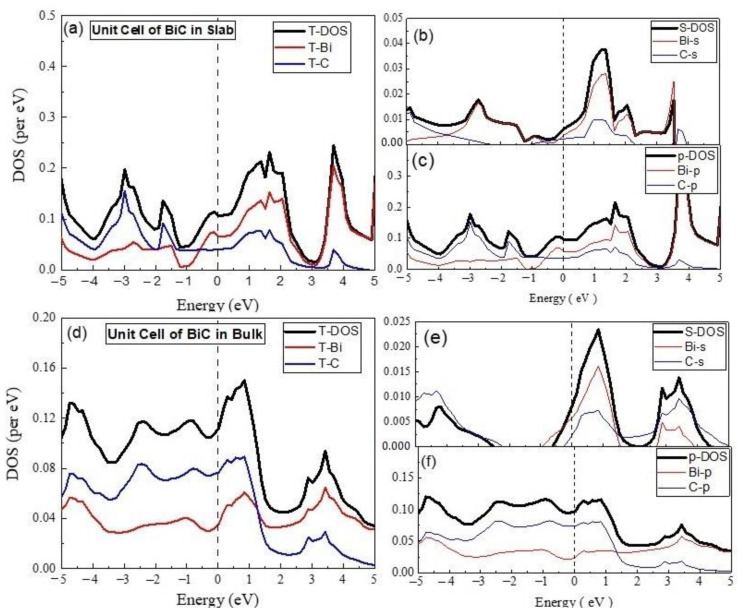
The calculated total density of states (TDOS) and partial density of states for BiC unit cell (**a**) TDOS of slab (**b**) s-DOS of slab (**c**) p-DOS of slab (**d**) TDOS of bulk (**e**) s-DOS of bulk (**f**) p-DOS of bulk. The Fermi level was adjusted at 0 eV.

The total density of states (TDOS) and partial density of states (PDOS) calculated for BiC unit cell in 2D periodicity are given in Figure 2.

The analysis of TDOS indicated the major contribution of the Bi states in the formation of valence band maxima (VBM), conduction band minima (CBM), and conduction band (CB), whereas the lower and deep parts of the valence band (VB) comprised the main involvement of the C states. Bi and C, with the respective valence shell configurations 6s^2^6p^3^ and 2s^2^2p^2^, are responsible for electronic structure of their compounds. Considering the electronic configurations, the calculated partial DOS involving the s and p states of the compounds are shown and analyzed. The observation of the s-DOS near the Fermi level revealed the greater contribution of Bi-s states in the valence band (VB), as well as in the conduction band (CB), in comparison to that of than of C-s states. However, in the case of the p-states, the trend was different, as the contribution of the Bi-p states dominated in CB, whereas those of the C-p states were higher in the VB. The involvement of both Bi-p, with C-p as the majority, and Bi-s, with C-s as the minority, states at the Fermi level were found, which points to the role of the sp hybrid states in the transport properties of compound BiC. The mentioned electronic structure indicated the metallic nature of BiC. The unit cell of BiC was periodically extended in 3D to examine the electronic properties of the materials. The calculated DOS are given in Figure 2.

Now, we are going to discuss the electronic properties of the BiC unit cell in 3D, as per calculated DOS given in Figure 2. The material remained metallic in nature; however, the composition of the states in the formation of the electronic structure changed. The observation of the s-DOS indicated that the Bi-s states dominated in CB, but the C-s states showed a comparatively major contribution in the VB. Contrary to the 2D case, the comparison of p-DOS revealed that the C-p states showed a greater contribution in VB, near the Fermi level and in the top portion of the CB. The comparison of TDOS indicated major parts of the C states in the formation of VB and CBM, as well as a Fermi level opposite of the 2D unit cell of BiC. However, the presence of sp hybrid states at the Fermi level were still observed in this case.

**Figure 3:**
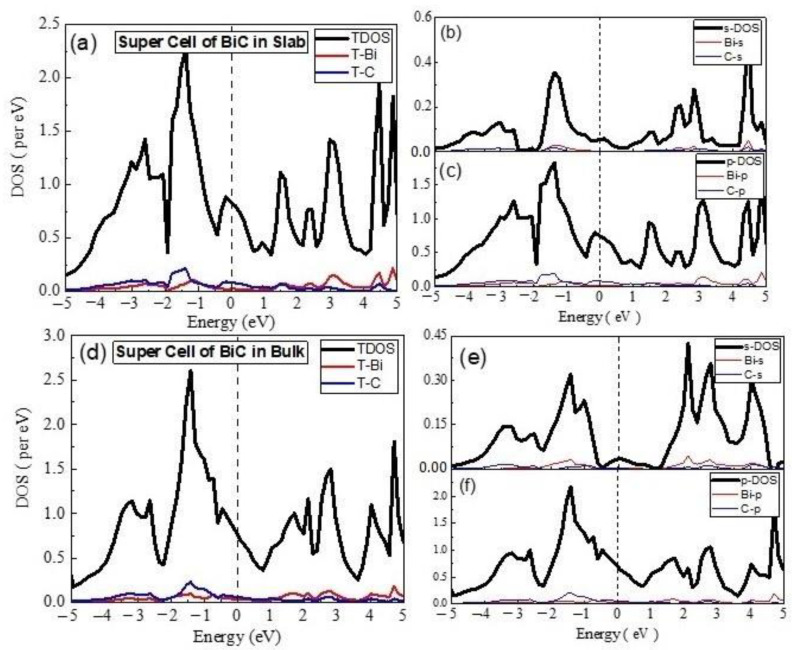
The calculated total density of states (TDOS) and partial density of states for BiC Super cell (**a**) TDOS of slab (**b**) s-DOS of slab (**c**) p-DOS of slab (**d**) TDOS of bulk (**e**) s-DOS of bulk (**f**) p-DOS of bulk. The Fermi level was adjusted at 0 eV.

The TDOS of 2 × 2 supercell in 2D (Figure 3) indicated the dominance of the C states in VB, Fermi level, and CBM, whereas the Bi states mainly contributed to the formation of deep CB, which showed deviation in behavior, when compared with the 2D unit cell of the material. The partial DOS points to major contribution of the C-p states and minor role of the Bi-p states, whereas the involvement of the s-states was very small. The material remained metallic in nature, with the involvement of sp hybrid states at the Fermi level. The 2 × 2 × 2 supercell of the material in 3D still showed a metallic nature, with major contributions of C-p states in the formation of VB at Fermi level and CBM, whereas the Bi-p states were dominant in the CB.

**(B)**  **Unit Cell of BiC_2_**

**Figure 4:**
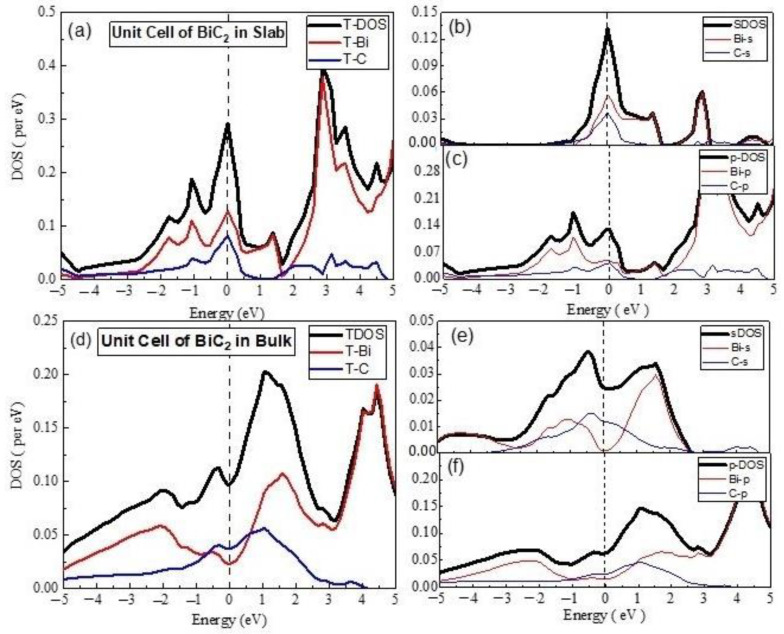
The calculated total density of states (TDOS) and partial density of states for BiC_2_ unit cell (**a**) TDOS of slab (**b**) s-DOS of slab (**c**) p-DOS of slab (**d**) TDOS of bulk (**e**) s-DOS of bulk (**f**) p-DOS of bulk. The Fermi level was adjusted at 0 eV.

**Figure 5:**
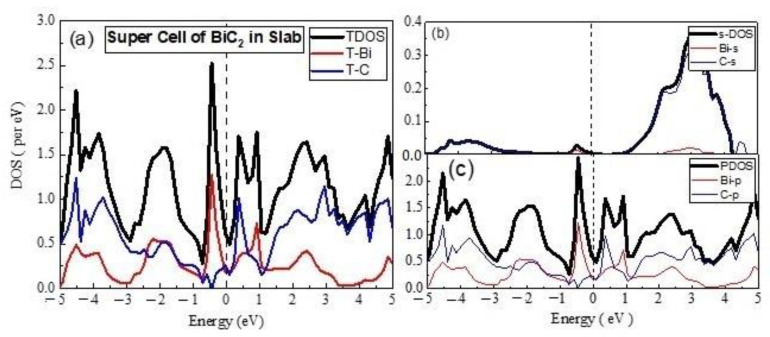
The calculated total density of states (TDOS) and partial density of states for BiC_2_ Super cell (**a**) TDOS of slab (**b**) s-DOS of slab (**c**) p-DOS of slab. The Fermi level was adjusted at 0 eV.

The TDOS and PDOS calculated for unit cell of BiC_2_ in 2D are given in Figure 4. The comparison of s-PDOS revealed the major contribution of the Bi-s states in the formation of VBM at Fermi level and the CB. On the other hand, the Bi-p states dominated, in comparison to that of C-p states, when TDOS or all PDOS were taken into account. The material was metallic in nature, with the sp hybrid states at the Fermi level. In the case of the BiC_2_ unit cell in 3D, the material was still metallic, with a comparative DOS and dominance of the p-states from both the Bi and C at the Fermi level. The contribution of the Bi-p states was rich in deep CB and deep VB, whereas the C-p states were bit higher at the Fermi level, as well as for VBM and CBM. The calculated DOS for the 2 × 2 supercell of BiC_2_ indicated the dominance of the p states in the electronic structure of the material, in such a way that the Bi-p states and the C-p states were rich in VBM (Figure 5). Both the Bi-p and C-p states equally contributed at the Fermi level, whereas the role of the Bi-s and C-s states were negligibly small.

**(C)**  **Unit Cell of BiC_3_**

**Figure 6:**
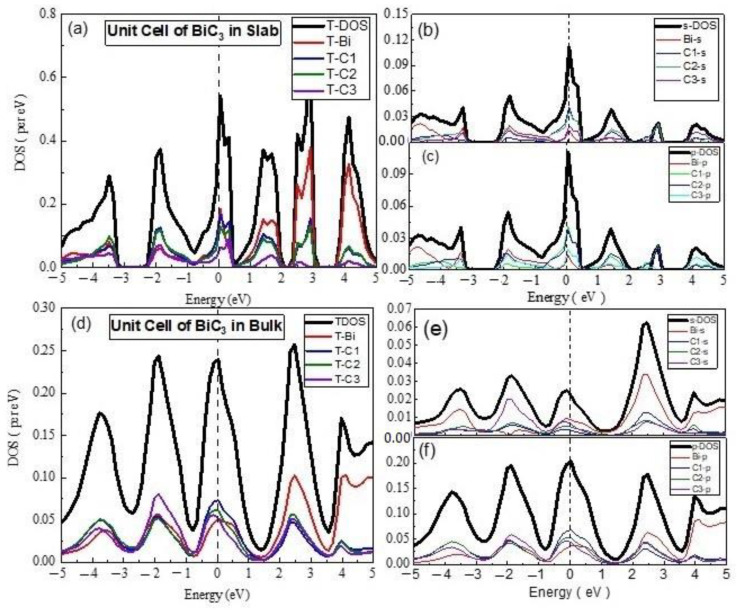
The calculated total density of states (TDOS) and partial density of states for mentioned atoms in BiC_3_ unit cell (**a**) TDOS of slab (**b**) s-DOS of slab (**c**) p-DOS of slab (**d**) TDOS of bulk (**e**) s-DOS of bulk (**f**) p-DOS of bulk. The Fermi level was adjusted at 0 eV.

In case of BiC_3_, the s-PDOS for the unit cell in 2D indicated a comparative contribution from the Bi-s and C-s states in VB and CB, but both equally contributed at the Fermi level (Figure 6). The survey of TDOS revealed nearly the same contribution from the Bi and C atoms in VB, but the dominant part of the Bi states in the CB, whereas both equally contributed at the Fermi level. The material was metallic in nature, with the sp hybrid orbitals taking part in the transport mechanism.

The 3D unit cell of BiC_3_ exhibited the rich involvement of the Bi-p and C-p states, in comparison to the s-states. The s-PDOS showed C-s rich states in VB; it showed Bi-s rich states in VB, whereas the Bi-s states were slightly higher at the Fermi level. On the other hand, the analysis of p-PDOS indicated the major contribution of the C-p states in VB and at the Fermi level, as well as and at the CBM, whereas the Bi-p states dominated in the deeper part of the VB. The situation of TDOS remains the same as those of the p-states, as mentioned earlier. The material was still metallic in nature.

The 2D supercell of BiC_3_ exhibited a similar electronic structure, but the contribution of the p-states were comparatively higher. The s-PDOS and p-PDOS revealed the dominant role of the Bi-s and Bi-p states, respectively. The states at the Fermi level were very low, which indicated that the material may be a narrow gap semiconductor, but in the current computational details, we assigned a metallic nature to BiC_3_. In the case of the 3D supercell of BiC_3_, the analysis of s-PDOS and p-PDOS pointed to the dominance of Bi-p states and the metallic nature of the material.

**(D)**  **Unit cell and Supercell of Bi_2_C_3_**

**Figure 7:**
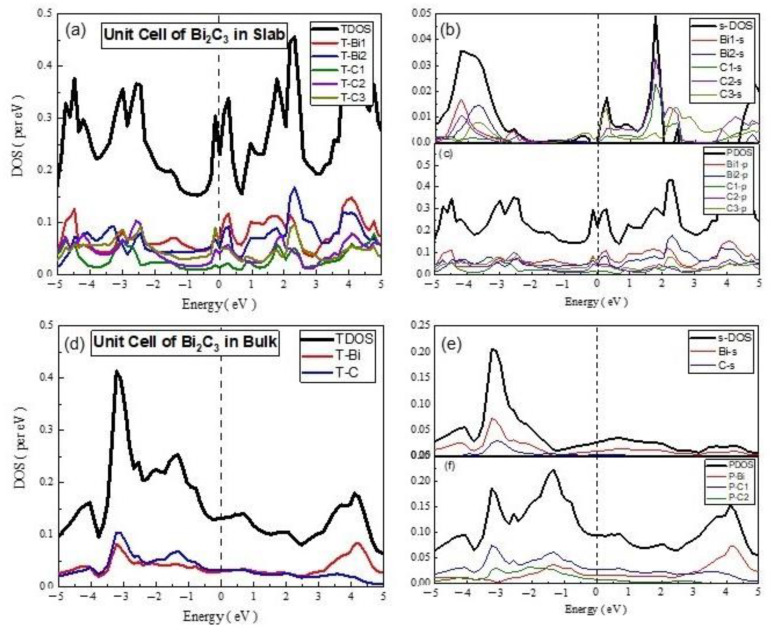
The calculated total density of states (TDOS) and partial density of states for mentioned atoms in Bi_2_C_3_ unit cell (**a**) TDOS of slab (**b**) s-DOS of slab (**c**) p-DOS of slab (**d**) TDOS of bulk (**e**) s-DOS of bulk (**f**) p-DOS of bulk. The Fermi level was adjusted at 0 eV.

The electronic properties of Bi_2_C_3_ were also studied by preparing its unit cells and supercells in 2D and 3D. The PDOS and TDOS calculated for 2D unit cell of Bi_2_C_3_ are given in Figure 7, which shows the dominant nature of the Bi-p and C-p states in the electronic structure of the material. The s-PDOS showed the dominance of the Bi-s states over the C-s states throughout. On the other hand, the p-PDOS and TDOS exhibited the major contribution of Bi-p in CBM and that of C-p in the formation of VBM. The material was metallic in nature, with sp hybrid states at the Fermi level.

**Figure 8:**
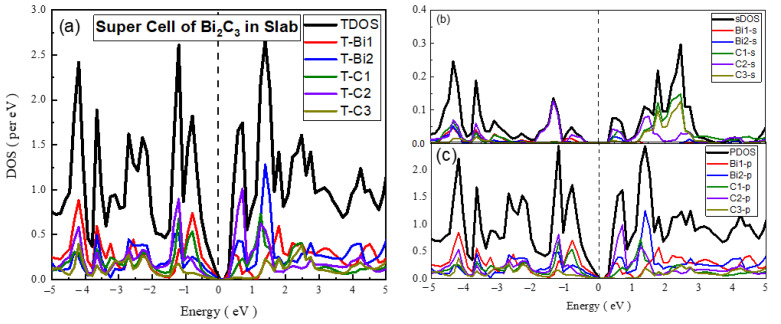
The calculated total density of states (TDOS) and partial density of states for the mentioned atoms in Bi_2_C_3_ super cell (**a**) TDOS of slab (**b**) s-DOS of slab (**c**) p-DOS of slab. The Fermi level was adjusted at 0 eV.

In the case of the 3D unit cell of Bi_2_C_3_, s-PDOS and p-PDOS showed nearly the same DOS of the s and p states of Bi and C (Figure 8). However, the comparison of s-PDOS revealed that the Bi-s states were richer than the C-s states, whereas the comparison of p-PDOS indicated that the C-p states were higher than those of the Bi-p states. The comparison of the TDOS exhibited nearly the same contribution of the Bi- and C-related states. Bi_2_C_3_ exhibited a semiconducting nature with a band gap of 0.305 eV, which was close to the reported values [43]. The analysis of partial DOS indicated that VBM comprised Bi-p states hybridized with C-p states. On the other hand, CBM was dominated by the C-p states, with a smaller contribution from the Bi-p states. These findings agree with the reported electronic properties of metal carbides [8,44].

**(E)**  **Super Cell of BiC_5_**

**Figure 9:**
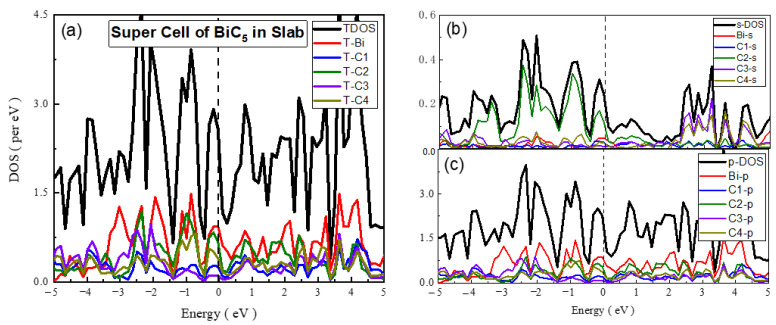
The calculated total density of states (TDOS) and partial density of states for mentioned atoms in BiC_5_ Super cell (**a**) TDOS of slab (**b**) s-DOS of slab (**c**) p-DOS of slab. The Fermi level was adjusted at 0 eV.

The calculated TDOS, SDOS, and PDOS of 3 × 3 super cells of BiC_5_ are shown in Figure 9. In the case of the s-DOS near the Fermi level, we observed that the major contributions in VB, as well as in CB, were due to the C4 atom, and they were minor for the C3 atom. While observing the graph of PDOS and TDOS, the major contribution in VB, as well as CB, was due to Bi atom, and it was minor for the C3 atom. From observing the TDOS, SDOS, and PDOS graphs, it is clear that total contribution in VB and CB was due to the Bi-p subshell. From the graphs, we indicated that there were no maximum and minima; additionally, overlapping occurred in VB and CB. Thus, the material is metallic.

**(F)**  **Unit cell of Bi_2_C_5_**

**Figure 10:**
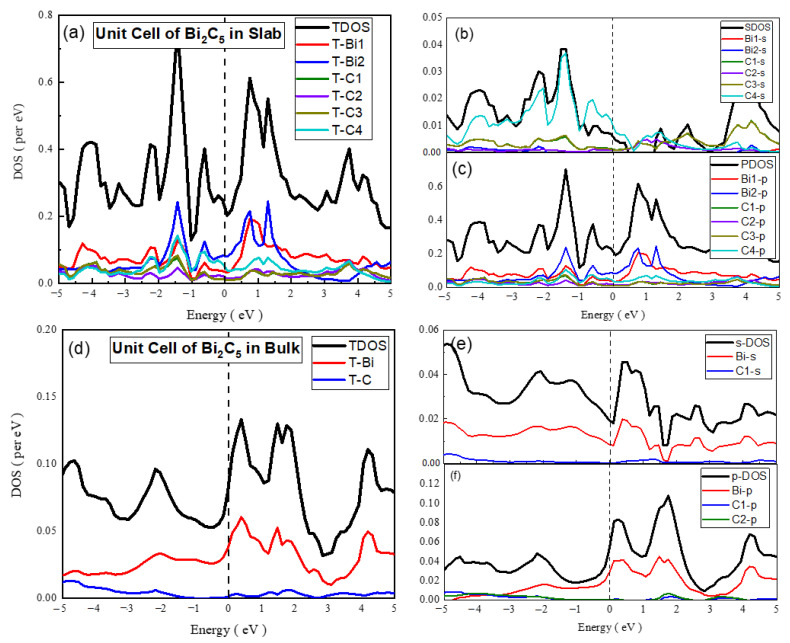
The calculated total density of states (TDOS) and partial density of states for mentioned atoms in Bi_2_C_5_ unit cell (**a**) TDOS of slab (**b**) s-DOS of slab (**c**) p-DOS of slab (**d**) TDOS of bulk (**e**) s-DOS of bulk (**f**) p-DOS of bulk. The Fermi level was adjusted at 0 eV.

The comparison of s-PDOS and p-PDOS calculated for the 2D unit cell points to the fact that the p-states dominated to define the electronic structure of Bi_2_C_5_ in such a way that the C-related states were found to be rich at the Fermi level. The analysis of s-PDOS indicated a richness of the C-s states in the formation of VB and CB, whereas the p-PDOS and TDOS revealed the dominance of the C-p states in the principal bands. In the case of the 3D unit cell of Bi_2_C_5_, the Bi-related states highly dominated, and the C states were suppressed in the electronic structures of the materials that appeared metallic in nature (Figure 10). The analysis of s-PDOS, in the case of the 2D Bi_2_C_5_ supercell, showed a major contribution of the Bi-s states in VB and CB, but the C-s states dominated at the Fermi level. However, the entire electronic structure of the material revealed the richness of the Bi-p states and metallic nature.

**(G)**  **Unit cell of BiC_5_**

From observing the TDOS, SDOS, and PDOS graphs, it is clear that total contribution in the VB and CB was due to the Bi-p subshell, and the material was found to be metallic [43].

## 4. Summary

The first principle calculations were performed to predict a series of compounds of Bi and C in the form of unit cell and super cell models of BiC, BiC_2_, BiC_3_, Bi_2_C_3_, BiC_5_, and Bi_2_C_5_ in two and three dimensions. The structural and electronic properties of the compounds were studied. The survey of the structural properties indicated that the bond length Bi-Bi remained almost the same for all compounds, whereas the bond lengths and orders for Bi-C and C-C showed variation. The symmetry of the materials appeared cubic. On the basis of charge analysis, it was found that Bi was cationic, whereas C was anionic. The entire materials were metallic, except 2D Bi_2_C_3_, which exhibited a semiconducting nature with a band gap of 0.305 eV. The mentioned Bi-C structures were reported, for the first time, in this paper; however, further investigations are required to investigate other possible structures in the series. Thus, this study is far from a complete structural search, and the prospects of more Bi_x_C_y_ compounds in different crystal structures are yet an open research area.

## Figures and Tables

**Table 1:** The optimized values of lattice Constant, Angles, Area/Volume, and Symmetry of Compounds in unit cell (U.C.) and supercell (S.C.) geometries.

Compounds	Lattice Constant	Lattice Angles	Area or Volume	Symmetry
U.C. of BiC in Slab	a = 3, b = 3		9 Å^2^	
U.C. of BiC in Bulk	a = 3, b = 3, c = 3	α = β = γ = 90°	27 Å^3^	Cubic
S.C. BiC in Slab	a = 6, b = 6		36 Å^2^	
S.C BiC in Bulk	a = 6, b = 6, c = 6	α = β = γ = 90°	216 Å^3^	Cubic
U.C. of BiC_2_ in Slab	a = 3, b = 3		9 Å^2^	
U.C. of BiC_2_ in Bulk	a = 3, b = 3, c = 3	α = β = γ = 90°	27 Å^3^	Cubic
S.C. of BiC_2_ of 2 × 2 Slab	a = 6, b = 6		36 Å^2^	
S.C. of BiC_2_ of 2 × 2 mono Slab	a = 6, b = 6		36 Å^2^	
S.C. of BiC_2_ of 4 × 4 mono Slab	a = 12, b = 12		144 Å^2^	
S.C. of BiC_2_ of 4 × 4 bi-layer Slab	a = 12, b = 12		144 Å^2^	
S.C. of BiC_2_ of 4 × 4 two bi-layer Slab	a = 12, b = 12		144 Å^2^	
U.C. of BiC_3_ in Slab	a = 3, b = 3		9 Å^2^	
U.C. of BiC_3_ in Bulk	a = 3, b = 3, c = 3	α = β = γ = 90°	27 Å^3^	Cubic
S.C. of BiC_3_ in Slab	a = 6, b = 6		36 Å^2^	
S.C. of BiC_3_ in Bulk	a = 6, b = 6, c = 6	α = β = γ = 90°	216 Å^3^	Cubic
U.C. of Bi_2_C_3_ in Slab	a = 3, b = 3		9 Å^2^	
U.C. of Bi_2_C_3_ in Bulk	a = 3, b = 3, c = 3	α = β = γ = 90°	27 Å^3^	Cubic
S.C. of Bi_2_C_3_ of Slab	a = 6, b = 6		36 Å^2^	
S.C. of Bi_2_C_3_ 10 × 10 Slab	a = 30, b = 30		900 Å^2^	
S.C. of Bi_2_C_3_ 10 × 10 2-Slab	a = 30, b = 30		900 Å^2^	
S.C. of BiC_5_ Slab	a = 9, b = 9		81 Å^2^	
U.C. of Bi_2_C_5_ in Slab	a = 3, b = 3		9 Å^2^	
U.C of Bi_2_C_5_ in Bulk	a = 3, b = 3, c = 3	α = β = γ = 90°	27 Å^3^	Cubic
U.C. of Bi_2_C_5_ in Slab	a = 9, b = 9		81 Å^2^	

## Data Availability

The data that support the findings of this study are available from the corresponding author upon reasonable request.
